# Effectiveness of a complex intervention to improve participation and activities in nursing home residents with joint contractures (JointConEval): study protocol of a multicentre cluster-randomised controlled trial [DRKS-ID:DRKS00015185]

**DOI:** 10.1186/s13063-019-3384-6

**Published:** 2019-05-29

**Authors:** Natalie Nguyen, Regina Thalhammer, Katrin Beutner, Susanne Saal, Ricarda Servaty, Hanna Klingshirn, Andrea Icks, Kristina Freyberg, Markus Vomhof, Ulrich Mansmann, Lien Le, Martin Müller, Gabriele Meyer

**Affiliations:** 1 Institute of Health and Nursing Science, Medical Faculty, Martin Luther University Halle-Wittenberg, Halle, Saale Germany; 2 Faculty of Applied Health and Social Sciences, Rosenheim Technical University of Applied Sciences, Hochschulstraße 1, 83024 Rosenheim, Germany; 3 Institute for Medical Information Processing, Biometry and Epidemiology, Faculty of Medicine, Ludwig-Maximilians-Universität München, Munich, Germany; 4 Institute for Health Services Research and Health Economics, Centre for Health and Society, Faculty of Medicine, Heinrich Heine University Düsseldorf, Düsseldorf, Germany; 5 Institute for Health Services Research and Health Economics, German Diabetes Centre, Düsseldorf, Germany; 6 Department of Medical Controlling, University Hospital Bonn, Bonn, Germany

**Keywords:** Joint contractures; Participation; Activities; Nursing homes; International Classification of Functioning, Disability and Health (ICF); Complex intervention; Multicentre cluster randomised controlled trial

## Abstract

**Background:**

Nursing home residents are frequently affected by joint contractures, which impacts their participation and daily activities. A complex intervention, the Participation Enabling Care in Nursing (PECAN), was previously developed and pilot tested to address their needs. Its effectiveness and safety will be evaluated in the present study.

**Methods/design:**

This multicentre cluster-randomised controlled trial will be conducted in 32 nursing homes spread over two regions of Germany. A total of 578 residents over 65 years old with joint contractures will be included. To compare the effect of the PECAN intervention with optimised standard care (usual care and an information session), randomisation will take place at a cluster level.

The individually tailored intervention was designed using the biopsychosocial model in the International Classification of Functioning, Disability and Health (ICF) to reduce activity limitations and participation restrictions resulting from existing joint contractures by addressing barriers and by strengthening supportive factors on an individual level and an organisational level.

The implementation strategy comprises a facilitators’ workshop, a peer mentoring approach including a peer mentor visit and telephone peer counselling, an in-house information event, an information session for the nursing team and a training session on collegial consultation for the facilitators. The in-house information event will also take place in the nursing homes of the control group. The primary outcome is the residents’ participation and activities after 12 months of follow-up as assessed using the PaArticular Scales. The secondary outcome is the residents’ quality of life. A cost-effectiveness analysis (costs per additional resident who experienced a decrease of ten points in the participation or activities subscale of the PaArticular Scales) and a cost–utility analysis (costs per additional quality adjusted life year) will be conducted. We will investigate barriers and facilitators in a comprehensive process evaluation.

**Discussion:**

We expect a clinically relevant improvement of participation and activities in residents with joint contractures. Our findings will provide important insights regarding participation in the situation of the affected individuals.

**Trial registration:**

DRKS, DRKS00015185. Registered on 1 August 2018. Universal Trial Number U1111–1218-1555. Registered on 26 July 2018.

**Electronic supplementary material:**

The online version of this article (10.1186/s13063-019-3384-6) contains supplementary material, which is available to authorized users.

## Background

Joint contractures are common in older frail people in geriatric care and are associated with pain, increased fall risk and decreased functional ability [1–3]. Hence, affected individuals experience limitations in their capacity to perform daily activities and to participate in social life, and may thus, require nursing care [1, 4]. Recent research has shown that activity limitations and participation restrictions in self-care, mobility or leisure activities are the most relevant aspects from the viewpoint both of older people with contractures living in nursing homes or in the community and of health-care experts [4–7]. Despite that, the structural impairment in terms of range of motion of the affected joint is still the most frequently reported outcome measure in clinical studies on joint contractures [8–10]. The number of older individuals affected varies because there is no standardised definition of joint contractures, the diagnostic criteria differ, and settings and populations vary [11]. To address and improve the living situation of older people with joint contractures in geriatric care, we carefully planned a set of research activities using the biopsychosocial model of the World Health Organization’s International Classification of Functioning, Disability and Health (ICF) [12].

The first project included the identification of aspects related to functioning and disability of those affected to develop a standardised instrument for assessing the impact of joint contractures [4–8, 11, 13–17]. Based on the findings of the first project, we developed and piloted the complex intervention Participation Enabling Care in Nursing (PECAN) to improve participation and activities in nursing home residents with joint contractures [10, 18, 19]. Methodologically, we follow the UK Medical Research Council (MRC) framework [20] for the systematic development and evaluation of complex interventions. The MRC framework has four key steps: (1) the development of the complex intervention based on the best available evidence, (2) an assessment of its feasibility, (3) an evaluation of its effectiveness and (4) its wider implementation [20]. We covered the first two steps in our previous studies, and the third step is the subject of this trial.

### Objectives

The present study aims to evaluate the PECAN intervention by assessing its effectiveness in improving the participation and activities in nursing home residents with joint contractures compared to a control group receiving optimised standard care, which includes an information session about joint contractures in addition to their usual care. We will assess activities and participation as the primary outcome measures using the subscales of a previously developed, standardised ICF-based instrument [17]. To understand the change process, we plan to investigate facilitating and impeding aspects of the implementation by accompanying the trial with a comprehensive process evaluation. Additionally, we will assess the cost-effectiveness (costs per additional resident who experienced a decrease of ten points on the participation or activities subscale of the PaArticular Scales) and the cost–utility [costs per additional quality adjusted life year (QALY)] by conducting a health economic evaluation alongside the controlled trial.

## Methods/design

### Design

**Fig. 1:**
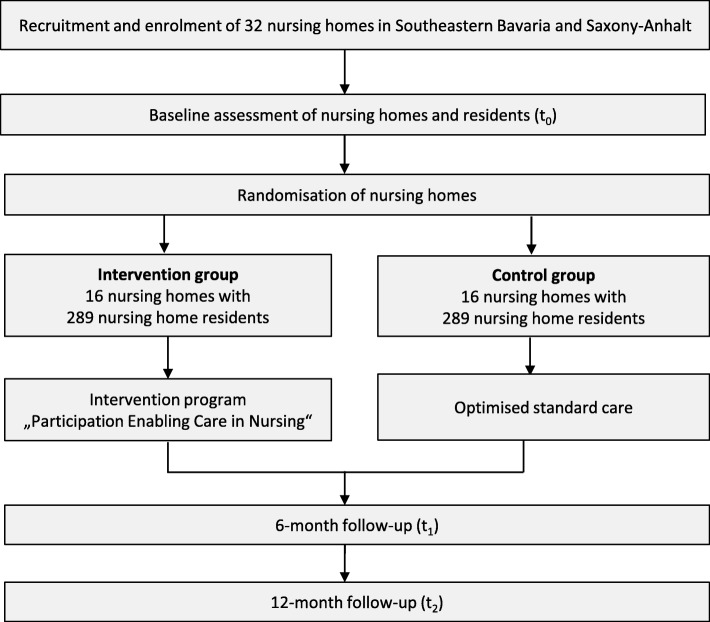
Flow of the clusters and participants through the trial

The study is a multicentre cluster-randomised controlled trial (c-RCT) with two parallel groups and a 1-year intervention period, with 578 residents in total (see Fig. 1). An intervention group with 16 nursing homes will receive the PECAN intervention and a control group with 16 nursing homes will receive optimised standard care, i.e. participants will receive information in the form of a presentation concerning the general aspects of joint contractures in geriatric care. Randomisation will be performed 1:1 on a cluster level. Outcome assessments will be performed at baseline with follow-ups at 6 and 12 months.

### Participants and recruitment

#### Sample size calculation

The study uses a composite primary outcome with two components. To avoid a false positive outcome rate above 5% (significance level), we apply a Bonferroni correction on each of the two components and choose the component-wise significance level as 2.5% (0.05/2 = 0.025). Based on the data from a pilot study, we consider both components as distributional comparable, assuming that both have the same variability. Therefore, the sample size considerations for each component follow the same arguments. Thus, it is sufficient to compute an explicit sample size for one component.

Using the experience of the pilot study (publication in preparation), recruitment of 15 to 20 individuals per cluster is feasible. Thus, the sample size calculation assumed a fixed cluster size of 15 individuals and a free number of clusters. Using pilot data, the intraclass correlation coefficient was estimated at 0.38. This resulted in an inflation factor of (1 + (15 – 1) × 0.38) = 6.32. The subscale variance observed in the pilot study was about 200 (standard deviation 14.14); the effect difference for the participation subscale between the standard and intervention groups was assumed to be 10 at t_2_. The size of one group in this c-RCT is *n* = 241 (38 × 6.32) if the test is two-sided on a significance level of 2.5% and with a power of 80%. This results in a total of 16 clusters per study group (241 / 15 = 16.1). To compensate for individuals terminating the study early due to death or moving, 15% more individuals will be included in the study, resulting in 30 clusters with a cluster size of 18 individuals and two clusters with 19 individuals, the total study size being 578 individuals.

Current experience shows that a few clusters will possibly fail to recruit the necessary number of participants. In this case, we will recruit more participants in other clusters to compensate for this. To maintain the statistical power of the study, the following rule will be applied: number of participants to be additionally recruited in other clusters = 1.5 × number of participants that some clusters fail to recruit.

#### Setting and eligibility criteria

The study will be performed in nursing homes in the German regions of south-eastern Bavaria and Saxony-Anhalt. The clusters are defined as nursing homes. We will include nursing homes in which at least 18 residents are affected by joint contractures. On the individual level, we will include nursing home residents aged 65 or older with present joint contractures in major joints that affect their daily life, who are likely to respond to the intervention, who can be mobilised into a sitting position, and who are able to understand and speak German. The joint contractures will be diagnosed by a physician, a skilled nurse or a physical or occupational therapist. In this study, they are defined as restricted joint mobility in at least one major joint (shoulder, elbow, wrist, hip, knee, or ankle). We will exclude nursing home residents receiving end-of-life care with limited life expectancy due to an advanced disease with a poor prognosis, and residents with congenital or idiopathic contractures i.e. Dupuytren’s contractures, plantar fibromatosis, and burn scar and other scar contractures.

#### Recruitment of clusters and study participants

Nursing homes will be recruited from convenience samples of interested or existing cooperation partners and from nursing home registers in each study region. They will be invited to participate in the study via electronic mail followed by a telephone call. The research staff will present the study to interested staff members on site and provide them with detailed information. Members of the advisory committees in the nursing homes will be included in all study activities as required by regional legislation.

After the director of the nursing home has provided written informed consent, the nursing home will be enrolled in the study. The head nurse will then identify eligible residents, and will supply them and, for cognitively impaired residents, also their legal guardians, with oral and written information about the study. Researchers from the study centres will be available for questions or to provide additional information in person or by phone. Eligible residents will then be reviewed by the management staff together with the research teams to ensure recruitment is standardised and to avoid heterogeneity in the sample. Subsequently, residents found to be suitable will be invited to participate in the study and informed consent will be obtained either from the resident or their legal guardian by the nursing home management. Consent can be withdrawn by residents and nursing homes at any time without stating a reason and without any disadvantages ensuing for either party.

#### Randomisation and allocation

Randomisation will be performed using stratified blocks. To ensure the intervention groups are balanced with respect to the location of the nursing homes, block randomisation will be performed taking two strata into account: (1) Halle (Saale) and (2) Rosenheim. Selection bias can be a problem in c-RCTs if participants are recruited after a cluster allocation has been implemented. Therefore, all clusters and participants will be recruited and assessed prior to randomisation. The clusters will be randomised by independent biostatisticians responsible for biometric supervision (UM and LL). Generating, operating and controlling the randomisation will follow the standard operating procedures of the biostatisticians’ institution and will be carried out by professionals who are not involved in trial activities at the nursing homes. Each of the nursing homes will be informed about its intervention allocation by fax by an independent data manager. Subsequently, the data manager will inform the study centres in Halle (Saale) and Rosenheim via fax about the allocation.

#### Blinding

Due to the characteristics of the intervention, it is not possible to blind the nursing staff, the nursing home residents or the researchers involved in delivering the intervention to the group allocation. However, the researchers and study assistants who collect the data at the different times and the biostatisticians who perform the data analysis will be blinded.

If an outcome assessor becomes unblinded, they will no longer collect data but will be replaced with another trained, blinded researcher. To estimate the success of blinding, the outcome assessors will be formally asked to guess the allocation of the study groups including a justification ; their responses will be compared with what would be expected by chance [21]. If data indicates that blinding was unsuccessful, we will replace the outcome assessor with another blinded researcher. 

### The PECAN intervention

**Fig. 2:**
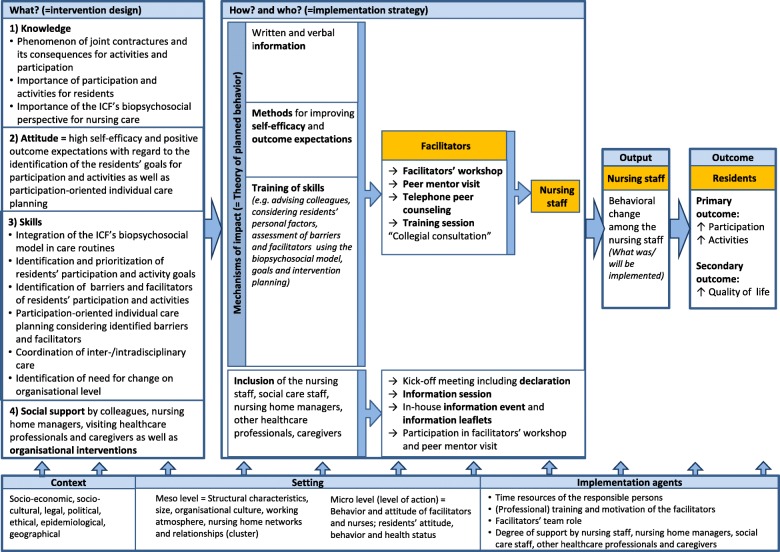
Logic model of the PECAN concept. ICF International Classification of Functioning, Disability and Health, PECAN Participation Enabling Care in Nursing

The experimental complex intervention PECAN was developed according to the MRC framework, and pilot tested in a previous project entitled JointConImprove [10, 18, 19]. We then systematically revised, updated and refined our logic model as well as the intervention and implementation components according to the results from the process evaluation of the pilot study (publication in preparation). The logical model for PECAN is outlined in Fig. 2. As Moore and Rhiannon [22] recommend, we integrated the contextual perspective in our model [23] and involved stakeholders in the development so that the underlying mechanisms were adequately addressed. Theoretically, PECAN was guided by the biopsychosocial model in the ICF, which provides a comprehensive framework for conceptualizing a person’s level of functioning as the dynamic interactions between their health status and environmental and personal factors, which can act as facilitators or barriers [12, 24].

The PECAN intervention aims to improve activities and participation in individuals with joint contractures by

- integrating the biopsychosocial model of the ICF into daily care activities
- identifying and prioritising the targets for activities and participation for each resident
- identifying the barriers and facilitators to the residents’ participation and activities
- implementing participation-oriented care planning, taking the identified barriers and facilitators into consideration.

Therefore, improvements in participation and activities may be achieved in four ways:

1. by improving impaired body functions and structures to allow activities and participation
2. by alleviating restrictions to daily activities through a resource-oriented promotion of activities to improve the residents’ autonomy in daily life
3. by considering the residents’ personal factors
4. by changing environmental factors to improve activities and participation.

To achieve the intervention goals, we will use an approach tailored to each individual.

Personal and environmental factors should be incorporated into each resident’s care plan and daily routine. In planning a resident’s personal goals, individual measures should follow a biographical approach that assesses the individual’s motives for participation. Environmental factors can be addressed by optimising the needs-based provision of adaptive technologies, medical aids and physical therapy, and by integrating family members or volunteers in organising activities during their visits.

On the organisational level, facilitators and barriers should be identified using a checklist with predefined criteria. In this way, the implementation of the PECAN intervention can be reviewed in terms of (1) the adaptations made to individual care plans for residents, (2) the dissemination of the principles of the intervention to co-workers, leaders and the public, (3) the interprofessional collaborations with social care assistants, therapists and social workers, and (4) the environmental factors for the site and the surrounding area.

#### Implementation strategy

**Fig. 3:**
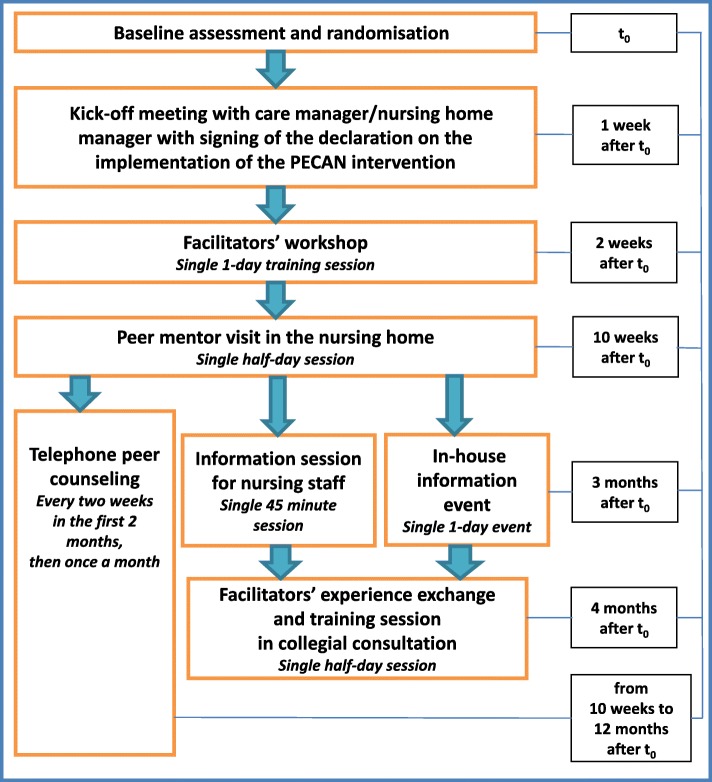
Overview of the implementation approach. PECAN Participation Enabling Care in Nursing

The implementation is based on the theory of planned behaviour [25], since our aim is to advance the professional attitudes of the nursing home staff such that their professional behaviour changes. Applying the theory of planned behaviour to predict or explain health-care professionals’ behaviour has been successful elsewhere [26, 27]. We chose a multifaceted facilitation approach in which we trained and supported nominated key nurses from the nursing homes, focusing on advancing their knowledge, attitudes and skills, and on utilising social support from nursing staff, management and other players. Facilitation is a concerted social process that focuses on evidence-informed practice change [28]. It has already been implemented effectively in nursing homes and in primary care [29–31]. Figure 3 is an overview of the implementation strategy.

##### Kick-off meeting

A kick-off meeting with the nursing home director and the head nurse will be scheduled shortly after the randomisation, at which the research team will provide detailed information about the PECAN concept, planned tasks for the implementation and required resources. The management’s commitment will be documented in a written declaration.

##### Facilitators’ workshop

A one-day-workshop for the nominated nurses will prepare them for their role as knowledge facilitators and change agents for the implementation of PECAN in their nursing homes. Facilitation depends upon the facilitator, who acts and enables others to implement a practice change [28]. There should be at least one facilitator per cluster, who will be appointed by the head nurse. As a prerequisite, the facilitators must have completed at least 3 years of vocational training in nursing or geriatric care.

The workshop will be run by the research team at the relevant study centre and covers the following content:

- information about the study and the facilitators’ future tasks
- current evidence about joint contractures, including their impact on participation, and their development, prevention and effective treatment
- strategies of and training in PECAN by applying the biopsychosocial model of the ICF to identify barriers and facilitators of the resident’s participation.

##### Peer mentoring

The peer mentoring includes a visit by an interdisciplinary team of peer mentors and continuous support by telephone for peer counselling. A study nurse from the research team at the relevant study centre will take on a mentor role and provide regular support to the facilitators during the intervention period.

##### Peer mentor visit

Our peer mentor visits are based on the concept of nursing peer review in which the care provided by nurses is systematically evaluated by a group of peers [32]. This tool helps to ensure the quality of nursing care through the application of evidence-based principles and fosters a continous learning culture of patient safety and best practice [32]. The half-day peer mentor visit will occur once during the intervention period to provide the facilitators with purposeful counselling and support. The head nurse and the nursing home directors will also be invited. The research team visiting the nursing homes will comprise an external peer expert with experience in geriatric care for residents with joint contractures and in change management, the mentor (study nurse) and an additional member of the research staff.

The peer experts will provide supervision at the individual level by reviewing the care plans of two participating residents. A structured tool will be used to assess patient-related issues and to guide the facilitators in planning the intervention. Moreover, any calls for action at the organisational level will be systematically reviewed using a checklist to identify action points in the implementation process. Finally, the next steps at the individual and organisational levels will be planned together, whereby the mentor will support the facilitators in implementing the PECAN intervention. Additional visits can be requested by the facilitators, which will be discussed during the telephone counselling sessions.

Additional material will be supplied, such as information pamphlets for relatives, legal guardians and health-care professionals. The facilitators will be given material e.g. motivational posters that they can use to promote the aims of PECAN in the nursing homes.

##### Telephone peer counselling

The facilitators will receive counselling from their mentor via regular phone calls at least once a month, starting after the peer mentor visit and lasting for nine months. The mentors will also be available for further advice during regular office hours. In each counselling session, the facilitator will give feedback of their experience with the implementation of the PECAN intervention, the tasks performed in the past weeks and their realisation of the implementation plan. The mentors will also counsel on communication issues within both the team and the organisation and answer technical questions on medical aids. The mentors will also be responsible for discussing any necessary organisational changes identified during the peer mentor visit or the telephone counselling with the nursing home managers.

##### In-house information event

A one-day in-house information event will be held in the nursing homes for residents, relatives, interested members of the public, volunteers and nursing home staff including social workers, visiting therapists, visiting physicians and advisory committee members. To create awareness of the project, we will supply written and oral information, geared to the different target groups, about the development and impact of joint contractures on participation, plus a basic overview of current evidence, details about the study and PECAN as well as suggestions for how the various individuals can support the implementation.

##### Information session for nursing staff

A brief information session of 45 min will be held by the research team for the nursing staff during a regular team meeting. It will provide information about PECAN, its aims, the tasks of the facilitators and how the nursing staff can support the intervention. The research team will illustrate the underlying principles using a short case vignette.

##### Facilitators’ experience exchange and training session on collegial consultation

Both PECAN and methods of collegial consultation [33] will be practised in a half-day training session by discussing actual cases (case vignettes). Members of the research team will train the facilitators on how to advise and coach their colleagues during the implementation. Furthermore, the facilitators will be able to exchange their experiences and discuss barriers and facilitators to implementation.

#### Modification and risk–benefit assessment

Possible benefits for the participants are improvements in activities or participation despite existing functional limitations due to joint contractures plus a reduction in the risk of further joint contractures. No modification of PECAN is planned, and no side effects, risks or complications are expected due to participation in the intervention. However, possible complications will be identified through the continuous close peer mentoring and reacted to promptly. In addition, the facilitators will be encouraged to talk directly to the relevant researchers, should problems arise. In the pilot study, there were no relevant differences between the intervention and control group regarding potential adverse events, such as the frequency of falls and fall-related fractures, nor was there any relevant increase in comparison to the baseline.

#### Control group

The nursing home residents in the control group will receive optimised standard care based on the usual care plus a brief in-house presentation of 45 min for nursing home staff, mainly nurses and additional care helpers. The session will cover information on the study, on the development of joint contractures, and on their relevance for daily life and the social participation of the affected individuals. It will also include a basic overview of current evidence, excluding intervention-related details.

To avoid contamination between the intervention and the control groups, the participating nursing homes will be obliged to refrain from actively disseminating intervention components to any other institutions, especially to other nursing homes in the control group. After the intervention period, nursing staff from the control group are given the opportunity to attend the workshop for facilitators and to receive the educational material and handbook free of charge.

### Outcome measures

Data for outcomes and other variables will be collected at three measurement points: at baseline before randomisation (t_0_), after 6 months (t_1_), and after 12 months (t_2_). Table 1 provides an overview of the different variables on an individual level, which includes the ratings of the nursing home residents. We will assess the cognitive status of the residents at each time point to decide if the outcome measurements will require a proxy rating, which the nursing staff will perform.

**Table 1:** Instruments and measures at resident level

Instrument	Outcome	Measurement point		
		t_0_	t_1_	t_2_
Primary outcomes				
PaArticular Scales, subscale participation [17]	Participation	X	X	X
PaArticular Scales, subscale activities [17]	Activities	X	X	X
Secondary outcomes				
EQ-5D-5L [34, 35]	Health-related quality of life	X	X	X
Additional data sources				
Medical records	Clinical data	X	X	X
DSS [36]	Cognitive impairment	X	X	X
CMAI [37, 38]	Behavioural symptoms	X	X	X
Clinical records	Falls and fall-related consequences	X	X	X
Clinical records	Physical restraints	X	X	X

*CMAI* Cohen–Mansfield Agitation Inventory, *DSS* Dementia Screening Scale, *EQ-5D-5L* EuroQol Five-Dimension Five-Level Instrument, *t*_*0*_ baseline before randomisation, *t*_*1*_ 6 months post-randomisation, *t*_*2*_ 12 months post-randomisation

#### Primary outcome

The initial objective of the project was to improve participation in nursing home residents with joint contractures. However, the results of the pilot study suggested that an improvement in participation is difficult to realise, especially within a population undergoing cognitive decline. Since activities and participation are related but independent constructs with no ranking, we decided that an improvement in any of the scales could be viewed as success. Therefore, the selected primary outcomes assess the nursing home residents’ participation and activities, which are measured with the two subscales of the ICF-based PaArticular Scales [17], a patient-centred self-reported measure. The PaArticular Scales were developed in a previous project using item response theory (Rasch analysis) based on a standard set of questions for joint contractures [15]. The instrument has two independent subscales. The participation subscale measures the impact of joint contractures on the participation of a person, i.e. the social perspective of functioning involving interactions with other people. It has 11 items on topics such as community life, sports, crafts and socialising. The activities subscale measures the impact on aspects of life related to mobility and self-care. It has 24 items on topics such as changing and maintaining body position, walking and moving, carrying, moving and handling objects, dressing and eating.

The scales were developed and validated in a sample of nursing home residents and patients in geriatric rehabilitation who were affected by joint contractures. The items were selected in a comprehensive standardised procedure in line with the suggestions for developing ICF core sets [39, 40]. The Rasch modelling approach to constructing scales based on items from the ICF has been shown to be valid in other studies [41, 42]. Before any analyses on the primary outcome are carried out, the psychometric properties of the PaArticular Scales will be re-evaluated in a Rasch-analysis of the data from the pilot study.

For each subscale, the nursing home resident or, if not possible, a proxy (nurse), assesses their existing difficulties for the different items on a scale ranging from 0 for “no problem”, 1 for “mild to moderate problem” and 2 for “severe problem” to 3 for “complete problem”. Additionally, an item is assigned “C” if the difficulty can be assigned without doubt to a cause other than the contracture. For both the participation subscale and the activity subscale, the scores are summed to give an initial ordinal raw score. This is transformed into an interval scaled score, ranging from 0 to 100, to allow comparisons at the interval level.

A mean decrease of ten points on the participation or activity subscale of the PaArticular Scales at t_2_ is considered as a clinically meaningful difference in favour of the PECAN intervention. This would represent a change from “complete problem” to “mild/moderate/severe problem” or from “mild/moderate/severe problem” to “no problem” in one of the patient-relevant items. The ten-point difference is also in line with the suggested distribution-based methods for defining a minimally important difference as at least 1/2 of the standard deviation [43], because the standard deviation in the baseline data from our pilot study is about 14, which is below 20.

In a recent review of the test–retest reliability of patient-centred self-reported measures for older people, the methodological quality of the PaArticular Scales was assessed as “fair” [44].

#### Secondary outcome

The secondary outcome is the nursing home residents’ quality of life. We will assess health-related quality of life at t_0_, t_1_ and t_2_, using the German version of the internationally established generic instrument the European Quality of Life Five-Dimension Five-Level Scale (EQ-5D-5L) [34, 35]. The residents can rate their health status using the following five dimensions: mobility, self-care, usual activities, pain/discomfort and anxiety/depression. Each dimension has five levels: no problems, slight problems, moderate problems, severe problems and extreme problems. Furthermore, a vertical visual analogue scale (EQ-VAS score) records a resident’s self-rated health from best to worst imaginable (range 0–100). In the corresponding proxy version, the nursing staff will be asked to rate how they think the residents would rate their own health-related quality of life, if they were able to communicate it.

#### Additional measures

We will collect further data for descriptive purposes (see Table 1). On an individual level, we will collect clinical data such as length of stay at baseline. In addition, we will collect grade of care, urinary and faecal incontinence, affected joints, provision of adaptive technologies, medical aids, physical therapy and occupational therapy at each time point. Additional characteristics will be collected from nurses’ assessments of the residents’ cognitive status and behavioural symptoms that are related to dementia.

A nurse will rate the cognitive status of each resident during the last month of the intervention using the Dementia Screening Scale (DSS) [36] to assess whether a proxy rating is required. The DSS contains the dimensions memory and orientation, giving a total of seven items each with a three-point scale (never, occasionally or always). The tallied responses result in an overall score with a range from 0 to 14, higher values indicating a greater degree of cognitive impairment. A proxy rating is performed at a cut-off point of 3.

The residents’ behavioural symptoms will be determined using a modified German version [45] of the Cohen–Mansfield Agitation Inventory (CMAI) [37, 38], as used in previous studies [46, 47]. The inventory consists of five symptom complexes: restlessness, verbal agitation, handling things inappropriately, negative attitude and aggression. Each complex is rated on a four-point Likert scale (never, once or twice, repeatedly, permanently) that assesses symptoms within the preceding four weeks.

On a cluster level, we will record the ownership of the nursing home (public, non-profit-organisation or private), its location and surroundings, the number of residents, the number and size of wards, the number of residents with joint contractures, staff mix and ratio, and the use of strategies for dealing with joint contractures or participation restrictions by interviewing the director of the nursing homes or head nurse at baseline.

To document adverse events that are potentially associated with the PECAN intervention, we will collect data on an individual level concerning the incidence of falls, fall-related consequences as well as the number and types of physical restraints used.

#### Data collection and management

All assessments will be performed on a paper-and-pencil basis by blinded members of the research team, who will be thoroughly trained in the data collection procedure.

Only pseudonymised data will be collected at the study centres in Halle (Saale) and Rosenheim, i.e. no personal data about the participants will be recorded or saved. The participating nursing homes will be allocated a pseudonymised number. To compare the outcomes from the measurement points, each participant will be assigned a code from a list, which will be stored separately and can be accessed only by the researchers at the study centres. The list will never be merged with the pseudonymised data and will be destroyed after the final validation of the analysis data set.

After each measurement point, the paper-based pseudonymised data at resident level from the study centres in Halle (Saale) and Rosenheim will be transferred to the biostatisticians’ institution, where the data entry and analysis will be performed by the data managers and biostatisticians. A copy of the questionnaires will remain safely stored at the respective study centres.

All answer to the questionnaires will be entered into a database and validated through double entry. The first data entry will take place at the latest 1 month after the different time points. Additionally, the data will be checked for inconsistency and completeness and its quality will be reported to the study centres.

The pseudonymised data will be stored on a secure computer. These data will be handled in accordance with the data protection declaration of the biometric institution. The server and all connected data arrays are in a limited access server room that is protected by a key card system and an alarm. The data will be encrypted and password-protected when being transferred between secure servers. Any hard-copy printouts, USB data versions or other removable media that are used to transfer protected health information will be destroyed after the transmission is complete.

Given the nature of the intervention, we do not expect either any serious safety concerns or an early termination of the study. There is, therefore, no necessity for a data monitoring committee. Moreover, we do not expect any serious adverse events. Thus, no interim analysis is planned, and there are no stopping rules for safety issues. However, we plan to monitor the data quality in a blinded manner every 6 months during our regular study meetings. Data quality (e.g. proportion of missing values) and organisational or logistic issues will be reviewed and discussed in these meetings. In this way, any necessary actions to improve data quality and study logistics can be made early enough.

### Statistical analysis

The primary outcomes are the nursing home residents’ participation and activity, measured with the subscales of the ICF-based PaArticular Scales [17]. The evaluation of the impact of joint contractures and the effect of PECAN will be based on an intention-to-treat approach with a significance level of 0.05, which results in a Bonferroni-adjusted significance level of 0.025 for a single test. The analyses will use a generalised linear mixed-effect regression model to compare changes in the scale between the treatment groups over a year. Each nursing home will be given a random intercept. The analysis will be adjusted by the individual baseline values.

#### Secondary analyses

The secondary outcome is the nursing home residents’ quality of life, which will be assessed at t_0_, t_1_ and t_2_ with the EQ-5D-5L [34, 35]. The analyses will also use a generalised linear mixed-effect regression model with EQ-5D-5L score changes over 1 year as the dependent variable, adjusted by the baseline value. Each nursing home will be given a random intercept.

The analyses of the primary and secondary outcomes will also allow for an additional adjustment for cluster and individual-specific confounding baseline variables such as age, gender, length of stay and grade of care at the individual level as well as ownership, number of residents, staff mix and ratio, and use of strategies for dealing with joint contractures or participation restrictions.

#### Further statistical issues

At each time point, if DSS ≥ 3, the outcome measurements will require a proxy rating. Sensitivity will be ascertained by removing these proxy ratings from the analysis. To investigate the impact of other influencing factors on the effect of PECAN, different subgroup analyses might be done. The necessity and feasibility of different subgroup analyses will be considered. The impact of missing values will be done by comparing complete case analyses with multiple imputation analyses.

### Process evaluation

**Fig. 4:**
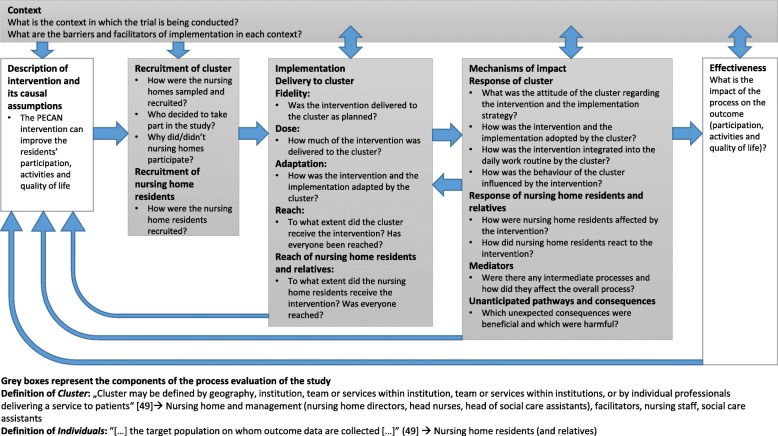
Process evaluation adapted from Grant et al. [49] for c-RCTs and from the MRC guidance [48]. c-RCT Cluster-randomised controlled trial, MRC UK Medical Research Council, PECAN Participation Enabling Care in Nursing

The process evaluation follows the MRC guidance for complex interventions [48]. The results will be used to understand the implementation of PECAN, causal mechanisms and contextual factors that influence participation, activity and quality of life [49]. Grant’s framework for designing process evaluations of c-RCTs will also be applied [49]. Along with the MRC guidance, this framework was adapted to make it appropriate for our study (see Fig. 4).

According to Moore et al. [48], the three focal points (implementation, mechanisms of impact and context) as well as their interaction with each other are crucial in a process evaluation. For the generalisability of the results, another central component of a process evaluation is the recruitment of clusters [49].

When evaluating recruitment, the implementation and the mechanisms of impact, we differentiate between the cluster level and the individual level. For the study, distinguishing between these levels in the process evaluation is meaningful because the PECAN intervention is not delivered directly to the residents, but via the cluster [49]. The aim of the PECAN intervention is to produce behavioural changes by the nursing staff in the clusters, which will ultimately affect the residents.

The process evaluation of the implementation considers the structures, resources and processes through which delivery succeeds [48]. This includes an assessment of the quality (fidelity) and the quantity (dose) of what was delivered and to what extent the PECAN intervention has reached the clusters and individuals. The evaluation also includes the adaptation of the PECAN intervention by the individual clusters [48]. To evaluate the mechanisms of impact, the intervention activities and how the clusters and individuals interact with the intervention activities will be considered. Additionally, mediators, unexpected processes and their consequences are recorded in this evaluation [48, 49].

The component context contains all the external factors that influence the delivery and the functionality of an intervention. Contextual factors can influence the effectiveness of the intervention both indirectly by shaping the implementation and directly by affecting the mechanisms of impact [48]. The evaluation can also highlight the relationships between the study processes and the primary and secondary outcomes [49]. In addition, differences in the effectiveness of the intervention at the different nursing homes can be explored.

According to Moore et al. [48], it is more useful to answer the most important questions (core questions) than trying to solve every question raised by a process evaluation. Therefore, it is necessary that the causal assumptions substantiating the intervention are known [48]. A mixed-methods approach will be pursued, including quantitative and qualitative methods of data collection and data analysis. Quantitative data will be analysed using descriptive statistics. Qualitative data from focus groups and semi-structured interviews will be tape-recorded and transcribed verbatim for analysis. These data and data from open-ended questions in the questionnaires will be analysed using content analysis [50] by two independent members of the research team.

The central questions in the process evaluation and the data collection procedures are listed in Fig. 4; a detailed overview is presented in Additional file 1.

### Health economic evaluation

The objective of the health economic evaluation is to estimate the cost-effectiveness of the PECAN intervention in terms of additional costs per additional resident who experienced a decrease of ten points on the participation or the activities subscale of the PaArticular Scales. Moreover, a cost–utility analysis will consider the additional costs required for each additional QALY induced by the intervention.

The economic evaluation will be performed from the perspective of the German social insurance system, which has statutory health insurance and long-term care insurance. Thus, an incremental cost-effectiveness ratio (ICER) will be calculated. This is defined as the difference in costs for the intervention and the control group divided by the difference in the number of nursing home residents who experienced a decrease of ten points in the participation or the activities subscale. Similarly, an incremental cost–utility ratio (ICUR) will be derived. This is the difference in costs divided by the difference in QALYs.

The primary outcome of the study will serve as the effect parameter in the cost-effectiveness analysis. Utility in the cost–utility analysis will be assessed by QALYs. These are based on health-related quality of life, which will be measured by the EQ-5D-5L and evaluated by a German tariff [51] to generate utility values.

Costs will be collected during the study for intervention-related components as well as for outcome-related components. In detail, resource use associated with the PECAN intervention (e.g. wage costs for the instructor associated with the facilitators' workshop and the opportunity costs of the trained nurses) will be derived from the study documentation. Costs explicitly associated with the study, such as data collection, will not be considered. For outcome-related components, we will retrospectively document for the previous 6 months the costs of physical therapy and occupational therapy at all three measurement points (t_0_, t_1_ and t_2_). Fall-related health-care utilisation will be documented retrospectively at baseline (t_0_) for the previous 12 months and at t_1_ and t_2_ for the previous 6 months. Furthermore, care levels and available medical aids will be recorded at each measurement point.

Health-care resource use due to the intervention and other reported health-care use will be multiplied by unit costs. Currently, there are no German guidelines for costing in an economic evaluation based on standard unit costs. Hence, health-care resource use will be valued by unit costs from published sources and official statistics for Germany (e.g. charges and rates from administrative databases and pharmacy retail prices).

For the health economic analysis, mean costs as well as cost differences between the intervention and the control group will be calculated as cluster averages. ICER and ICUR will be calculated and the non-parametric bootstrap method will be employed to generate confidence intervals around the ICER and ICUR estimates [52, 53]. Uncertainty surrounding the ICER will also be presented on the cost-effectiveness plane [54, 55] and as a cost-effectiveness acceptability curve [56, 57].

### Quality assurance

The study will be planned, implemented and evaluated in accordance with the principles of good clinical practice [58] and the Declaration of Helsinki [59]. This study was registered with the publicly accessible German Clinical Trials Register (DRKS) under the ID DRKS00015185 before any study participants were enrolled. This study protocol will be published in a peer-reviewed journal and it will follow the most recent Standard Protocol Items: Recommendations for Interventional Trials (SPIRIT) statement [21, 60] and applicable criteria from the template for intervention description and replication (TIDieR) [61]. The corresponding checklists are presented in Additional files 2 and 3. To ensure safety, a detailed quality assurance plan will be developed. This plan will contain the detailed objectives of the study parts; will summarise the monitoring methods for data collection, validation and reporting; will state the methods for response monitoring; and will provide a schedule for field audits and reporting procedures. Standard operating procedures and an auditing plan will also be included. Standard operating procedures will be developed to provide the research staff with instructions for collecting, handling, reporting and evaluating the data. Furthermore, a scientific advisory board of at least three experts from relevant professions will be convened to supervise the trial.

To ensure they are widely disseminated, we will publish the main study outcomes in peer-reviewed scientific journals and will present the results at scientific conferences. All results will be reported in accordance with the extension of the Consolidated Standards of Reporting Trials (CONSORT) for cluster trials [62], the revised guideline in the Criteria for Reporting the Development and Evaluation of Complex Interventions in health care [63] and the TIDieR checklist [61]. All trial information will be freely available via the trial homepage (https://bewegung-verbindet.de/english-version/). The homepage contains general information about completed and ongoing projects as well as German and English publications, which are aimed at researchers, clinicians, nursing staff, health-care providers and consumers.

### Ethical and legal considerations

The research staff are obliged (1) to ensure that the subjects involved in the research have opportunities to address any sense of burden and (2) to maintain their well-being. The principal investigators will inform the ethic committees immediately of any changes in the study protocol, of any expected or unexpected serious events, or of the early termination of the study.

Prior to inclusion in the study, all nursing home directors, all head nurses and all available residents or their legal guardians will be informed orally and in writing about the nature, objectives, expected benefits and potential risks of the study, then given sufficient time and opportunity to decide whether to participate and to clarify open questions before informed consent is obtained.

The nursing homes and residents or their legal guardians will be informed that all study-related data will be stored in pseudonymised form and will be used only for scientific data analysis. All study data will be kept in a locked archive for ten years at the study centres after completion of the trial and will then be deleted. Furthermore, the EU General Data Protection Regulation will apply, and all participants will be explicitly informed about their rights. In the event of any violations of ethical standards with respect to the medical care of the residents or research-related activities within the study, a standardised protocol will be closely followed, supervised and executed by the study coordinator (MM).

## Discussion

In this c-RCT, we aim to evaluate the effectiveness of the complex PECAN intervention to improve participation and activities of older nursing home residents with joint contractures. We will use a customised approach to facilitate the realisation of the residents’ individual aims for participation in their daily lives. To enable the effective improvement of activities and participation, our intervention includes components at an individual level and an organisational level.

We aim to advance the nursing staff’s professional attitude and thus, support the implementation by underlining the influence of contractures on participation, autonomy and consequently, on the quality of life of impacted residents and by empowering the staff to apply PECAN.

Due to our experiences gained from the pilot study, we will focus specifically on improving the delivery of the intervention to residents and nursing staff. The rigorous process evaluation will ensure that the implementation process is supervised. It will ensure the safety of the PECAN intervention and it will allow us to adjust components or strategies, if necessary.

By including contextual factors at different levels in our logic model, we will be able to collect and observe influencing aspects systematically, e.g. new laws and directives regarding quality indicators for nursing home care will be included in the legal context.

We expect to see an improvement in the residents' activities or participation or both due to the carefully developed and tested PECAN intervention and no safety-related adverse effects. If proven successful, we will transform the intervention protocol into a training course for skilled nurses, which will be taught at advanced training institutes of the universities in Halle (Saale) and Rosenheim. Our findings will provide important insights into the social participation of older people with joint contractures.

## Additional files

Additional file 1: **Table S1.** Overview of the process evaluation. (DOCX 36 kb)

Additional file 2: SPIRIT 2013 checklist: recommended items to be addressed in a clinical trial protocol and related documents. (DOCX 51 kb)

Additional file 3: The TIDieR (Template for Intervention Description and Replication) Checklist. (DOCX 32 kb)

## Abbreviations

CMAI: Cohen–Mansfield Agitation Inventory
CONSORT: Consolidated Standards of Reporting Trials
c-RCT: Cluster-randomised controlled trial
DRKS: German Clinicals Trials Register
DSS: Dementia Screening Scale
EQ VAS: EuroQol Visual Analogue Scale
EQ-5D-5L: EuroQol Five-Dimension Five-Level Instrument
ICER: Incremental cost-effectiveness ratio
ICF: International Classification of Functioning, Disability and Health
ICUR: Incremental cost–utility ratio
MRC: UK Medical Research Council
PECAN: Participation Enabling Care in Nursing
QALY: Quality adjusted life year
SPIRIT: Standard Protocol Items: Recommendations for Interventional Trials
TIDieR: Template for intervention description and replication
