# Supplementary material for: Effectiveness of a complex intervention to improve participation and activities in nursing home residents with joint contractures (JointConEval): study protocol of a multicentre cluster-randomised controlled trial [DRKS-ID:DRKS00015185]
Source: Trials. 2019 May 29;20:305. doi: 10.1186/s13063-019-3384-6 (PMC6542100; doi:10.1186/s13063-019-3384-6)
Supplement: Supplementary file 1 — Table S1. Overview of the process evaluation. (DOCX 36 kb) [file 13063_2019_3384_MOESM1_ESM.docx]

Additional file 1

Table S1 Overview of the process evaluation

| **Measurement point** | **Domain** | **Assessment and Focus** | **Informant of data collection** | **Study arm** |
| --- | --- | --- | --- | --- |
| During recruitment process | - Recruitment of nursing homes | Standardised documentation forms:   - Evaluation of the recruitment procedure - Reasons for participation/non-participation | Researcher | IG/CG |
| During recruitment process | - Recruitment of nursing home residents | Standardised documentation forms:   - Evaluation of the recruitment procedure - Reasons for participation/non-participation | Researcher | IG/CG |
| Baseline t0 | - Response of nursing staff and social care assistants | Standardised questionnaire:   - Attitude regarding activities and participation - Integration of activities and participation in daily care - Organisational culture with the D-OCAI^[[1]](#footnote-1)^ | Nursing staff/Nursing students/ Nursing assistants/Social care assistants | IG/CG |
|  | - Response of management - Context | Standardised questionnaire:   - General data of the cluster - Presence of concepts to address participation and/or activities - Organisational culture with the D-OCAI | Nursing home directors/Head nurses/Head of social care assistants | IG/CG |
| Kick-off meeting | - Delivery to management | Standardised documentation forms:   - Evaluation by researcher | Researcher | IG |
| Facilitators’ workshop | - Response of facilitators | Standardised questionnaires - Pre/Post:   - Integration of activities and participation in daily care - Attitude regarding participation - Social support by other staff/persons - Organisational culture with the D-OCAI - Satisfaction with the workshop (Post) - Expectation regarding implementation (Post) | Facilitators | IG |
|  | - Delivery to facilitators | Standardised questionnaire:   - Evaluation by trainer | Researcher | IG |
| Peer mentor visit | - Delivery to cluster - Delivery to individuals - Response of cluster - Response of nursing home residents - Mediators - Unanticipated pathways and consequences | Standardised documentation forms:   - Documentation case conferences - Evaluation by study nurse | Study nurse | IG |
| Telephone peer counselling | - Delivery to cluster - Delivery to individuals - Response of cluster - Response of nursing home residents - Mediators - Unanticipated pathways and consequences | Standardised documentation forms:   - Documentation case conferences | Study nurse | IG |
| Facilitator experience exchange and training session | - Response of facilitators | Standardised questionnaire:   - Satisfaction with the training - Expectations regarding implementation | Facilitators | IG |
|  | - Delivery to facilitators | Standardised questionnaire:   - Evaluation by trainer | Researcher | IG |
| Information session | - Delivery to nursing staff and social care assistants | Standardised documentation forms:   - Evaluation by researcher | Researcher | IG |
| In-house information event | - Delivery to cluster - Delivery to individuals | Standardised documentation forms:   - Evaluation by researcher | Researcher | IG |
| Brief in-house presentation | - Optimised standard care | Standardised documentation forms:   - Evaluation by researcher | Researcher | CG |
| t1 (6 months after t0) | - Response of nursing staff and social care assistants | Standardised questionnaire:   - Attitude regarding participation - Integration of activities and participation in daily care - Organisational culture with the D-OCAI | Nursing staff/Nursing students/Nursing assistants/Social care assistants | IG/CG |
|  | - Response of management - Context | Standardised questionnaire:   - Presence (and changes) of concepts to address participation and/or activities - Organisational culture with the D-OCAI | Nursing home directors/Head nurses/Head of social care assistants | IG/CG |
|  | - Response of facilitators - Context | Standardised questionnaire:   - Integration of activities and participation in daily care - Attitude regarding participation - Social support by other staff/persons - Satisfaction with the implementation and support by study team - Organisational culture with the D-OCAI | Facilitators | IG |
| t2 (12 months after t0) | - Response of nursing staff and social care assistants | Standardised questionnaire:   - Attitude regarding activities and participation - Integration of activities and participation in daily care - Organisational culture with the D-OCAI | Nursing staff/Nursing students/ Nursing assistants/Social care assistants | IG/CG |
|  | - Response of management - Context - Mediators - Unanticipated pathways and consequences | Standardised questionnaire - cluster:   - Presence (and changes) of concepts to address participation and/or activities - Organisational culture with the D-OCAI | Nursing home directors/Head nurses/Head of social care assistants | IG/CG |
|  | - Response of relatives | 48 guided semi-structured telephone interviews, 3 persons/cluster:   - Experience within the implementation process - Changes in the cluster/individuals | Relatives | IG |
|  | - Response of nursing staff - Response of nursing home residents - Mediators - Unanticipated pathways and consequences | 16 guided semi-structured focus group interviews, 4-5 nurses/cluster:   - Experience within the implementation process - Changes in the cluster/individuals | Nursing staff | IG |
|  | - Delivery to nursing home residents | Standardised questionnaire:   - Attitude regarding activities/participation - Integration of activities and participation in daily care - Organisational culture with the D-OCAI | Nursing staff | IG/CG |
| Support facilitators t2 | - Response of facilitators - Context | Standardised questionnaire:   - Integration of activities and participation in daily care - Attitude regarding activities and participation - Social support by other staff/persons - Satisfaction with the implementation and support by study team - Organisational culture with the D-OCAI | Facilitators | IG |
|  | - Response of facilitators - Response of nursing home residents - Mediators - Unanticipated pathways and consequences | 4 guided semi-structured focus group interviews:   - Experience within the implementation process - Changes in the cluster/individuals | Facilitators | IG |

1. German version of the “Organizational Culture Assessment Instrument” derived from the “Competing Values Framework” [↑](#footnote-ref-1)
